# Staff experiences of encountering and treating outpatients with substance use disorder in the psychiatric context: a qualitative study

**DOI:** 10.1186/s13722-021-00235-9

**Published:** 2021-05-10

**Authors:** Elisabeth Petersén, Anna Thurang, Anne H. Berman

**Affiliations:** 1 Centre for Psychiatry Research, Department of Clinical Neuroscience, Karolinska Institutet, & Stockholm Health Care Services, Region Stockholm, Stockholm, Sweden; 2 Department of Psychology, Uppsala University, Uppsala, Sweden

**Keywords:** Substance use disorder, Comorbidity, Outpatient psychiatry, Staff attitudes, Addiction treatment services

## Abstract

**Background:**

High comorbidity exists between mental illness and substance use disorders (SUD). Patients in psychiatry living with problematic alcohol or drug consumption can experience a sense of exclusion, where seeking help for SUD can be perceived as stigmatizing. The aim of this study is to illuminate staff experiences of encountering patients with SUD within the psychiatric outpatient context.

**Methods:**

The study was exploratory, with a qualitative design. Interviews with outpatient psychiatry managers and focus groups with clinical staff focused on the experience of encountering patients with SUD. Data were evaluated using content analysis inspired by phenomenological-hermeneutic methodology.

**Results:**

Three themes were identified and each illuminated by two sub-themes. *Bridging the organizational gap* included sub-themes of having an established collaboration and facing difficulties in the collaboration; *Having beliefs about the patient you encounter* included sub-themes of working with patients who are exposed to prejudicial thoughts and expressing prejudicial thoughts about the patient. *Striving to achieve a therapeutic alliance* included sub-themes of having a feeling of developing together and supporting the patient towards recovery.

**Conclusion:**

A life-world perspective, used to interpret results, indicated that caring for patients with SUD in psychiatry was perceived as difficult, where collaboration between psychiatry and addiction care was often experienced as problematic. Based on these findings, we believe that the current gap between the psychiatry and addiction care could be reduced to some extent by offering patients digital treatment for SUD. In this way, patients could remain under the care of their regular psychiatric clinic without having to physically visit SUD services. Thus, a virtual bridge could be established to bring psychiatry and addiction care closer to each other for the patients’ benefit.

**Supplementary Information:**

The online version contains supplementary material available at 10.1186/s13722-021-00235-9.

## Background

The co-occurrence of mental illness and substance use disorder (SUD) is widely prevalent [1]. Among patients seeking psychiatric care, previous studies have shown that approximately 50% of people who have been diagnosed with mental illness at some point in their lives have also been diagnosed with SUD [2, 3]. By offering treatment for mental illness and addiction in combination, patient outcomes can be improved [4]. However, additional challenges face treatment providers, for whom it is more difficult to help patients with comorbidity than those with either psychiatric or substance use problems [5]. The clinical picture is further complicated by the problems that patients with comorbidity often show in other areas, such as finance, housing and social contacts. These patients therefore often receive interventions from several different caregivers including social services [6]. This happens despite research showing that integrated treatment, addressing all the patient's conditions, can lead to more consistent improvement in treatment outcomes [7].

Although offering patients integrated treatment is a promising solution [8], engaging patients in such treatment is challenging. Patients’ experience of stigma may lead to poor adherence within treatment as well as early dropout [9]. Several factors have been found to influence patients’ response to treatment. For instance, the healthcare professional-patient relationship has been found to greatly influence the patient's chance of responding positively to rehabilitation and treatment offered for substance use [10]. Furthermore, it is important to tailor treatment measures to the individual's understanding of their own capacity for change and their needs, which may vary by gender [11]. Patients’ engagement can also be related to their inner motivation to change, which depends on their personal experiences, physical health or lack of experienced ability to manage their life situation [12, 13].

In addition to patients being motivated, the staff need to be open to having a dialogue about substance use. Healthcare staff can experience an overall fear of caring for patients with mental illness, caused by an insufficient understanding of the patient's illness, which may lead to catastrophizing thoughts about what the patient group could do to staff and other patients [14], 15. Nurses who care for patients with SUD in addiction clinics have chosen to work with this patient group and are familiar with the complexity of the patients’ problems. Nurses working at clinics other than addiction clinics have stated that they may experience fear in connection with the care of these patients as they feel threatened and uncomfortable, particularly when the patients become aggressive [16, 17]. A study by Tsai et al. [18] found that although nurses perceived a good relationship with the patient as a prerequisite for discussing the patient's alcohol habits, half of the nurses were afraid that the relationship would deteriorate if they asked the patients about their alcohol habits [18]. Staff attitudes are also an important factor for treatment effectiveness among patients with substance use disorders [19]. Physicians' attitudes have proved more negative towards patients with substance use disorders, with and without schizophrenia, than towards patients who had only a schizophrenia diagnosis or a severe depressive disorder [20]. Furthermore, psychiatry residents have been found to show discriminating attitudes toward individuals with comorbid substance use disorders and mental illness. These attitudes seemed to deteriorate over time [21]. Nonetheless, such attitudes have proved responsive to education. For instance, attitudes among medical residents improved after an online training module [22]. Also, research among general practitioners and nurses in primary care suggests that having enough information and knowledge about substance use disorder is essential for staff to effectively support patients in the behavior change process [23].

In the present study, staff at psychiatric outpatient clinics with different specializations were interviewed about their experiences of encountering patients with a psychiatric diagnosis who have co-occurring problematic substance use. The substance use in this case does not necessarily mean that there is a diagnosis of substance use disorder, so these patients might not fall under the umbrella term of “dual diagnosis”. Whether or not a diagnosis of SUD has been determined, patients with mental health issues, substance use and other problems present a complex clinical picture. Patient-provider interactions are complicated by factors residing in the patient, as well as attitudes among staff. Progress in identifying and delivering effective treatment for comorbid conditions requires a deeper understanding of the triangular relationship between health professionals working in psychiatry, addiction care professionals, and the patient. Therefore, this study aims to illuminate health professionals’ experience of encountering patients with SUD within the psychiatric outpatient context.

## Methods

### Design

This was an exploratory study with a qualitative design. Data from interviews with outpatient psychiatry managers and staff focus groups were evaluated using content analysis inspired by phenomenological-hermeneutic methodology, where the purpose was to illuminate a deeper meaning of the experience of encountering patients with problematic substance.

### Setting

Data were collected in 2013–2015. Two pilot interviews were conducted in 2013, one with a psychiatry clinic staff member and one with a clinic manager in Stockholm, Sweden. These interviews were conducted at the Center for Psychiatry Research, Karolinska Institutet. After the pilot interviews, eight clinic managers in psychiatry were interviewed individually. In connection with these interviews, the managers were asked if it would be possible to return to the unit for a focus group interview with the staff. Focus group interviews were then conducted at three units, in a separate room where there was minimal risk of being disturbed.

### Context

In Sweden, treatment for substance use disorders and psychiatry is divided between two different organizational umbrella instances – regions and municipalities. The 21 regions coordinate healthcare services, where psychiatry clinics treat patients for diagnosed psychiatric disorders, while addiction services treat patients for diagnosed substance use disorders. The 290 municipalities coordinate social welfare services, where treatment units offer psychosocial treatment for substance use disorders, separately from healthcare services. Two different sets of legislation govern the regional and municipal services, with healthcare services in the regions (in this case, psychiatry and addiction) governed by the Health and Medical Services Act (1982:763), while social welfare services in the municipalities are governed by The Social Services Act (1980:620). Although municipalities and regions are legally obliged to collaborate in treating individuals with SUD, the extent of cooperation can vary greatly between different regions and municipalities. This creates an inequity of care for patients all over the country. More specifically, it can be challenging for those with a single addiction disorder, who can be referred both to regional and municipal treatment units. Also, those with co-occurring conditions may be referred for help with SUD to both regional and municipal units, as well as being treated in parallel for psychiatric issues within regional psychiatric care. The division between psychiatric and addiction services, complicated by the organizational divisions between regions and municipalities for addiction care, generates challenges for staff and patients that are the focus of this article.

### Sampling procedure

Study participants were recruited from two different populations within outpatient psychiatric clinics in the Stockholm region. The inclusion criterion in the first population was employment as a manager, and the inclusion criterion for the second population was employment as a treatment provider, with some kind of treatment contact with patients. Managers were originally identified as willing to participate in an interview in a national survey of managers and staff in psychiatry on the same topic [24] and were asked via e-mail whether they were willing to participate in an individual interview. Those who accepted were interviewed for this study. The focus group interviews in this study were arranged after the managers who had participated in an individual interview agreed to facilitate staff participation in focus groups.

### Data collection

Data were collected through semi-structured interviews, in both the individual and focus group contexts. The individual interviews took place in a psychosis clinic, borderline unit, ADHD clinic, general psychiatric clinics, affective clinic, and in specialized psychiatry clinics. The focus group interviews took place at a psychosis clinic in a suburb of Stockholm, a general psychiatric clinic in a suburb of Stockholm and at an ADHD clinic in the region of Stockholm. Both the individual and focus group interviews were carried out by two interviewers, one who led the interview and one who observed and asked follow-up questions as needed. Individual interviews lasted between 36 and 54 min and focus group interviews lasted between 65 and 80 min; individual interviews were conducted between 2014 and 2015 and focus group interviews in the spring of 2015. See Additional files for individual and focus group interview guides. The individual and focus group interviews provided a large quantity of material altogether. In order to gain a deeper understanding of the phenomena discussed in both types of interviews, we divided the analysis into two parts, where this article focuses on the actual experience of encountering patients with SUD; a forthcoming article will focus on exploring staff thoughts on the prospect of working with digital interventions. See Additional file 1 for the interview guide for the individual interviews with psychiatry clinic managers; see Additional file 2 for the interview guide for focus groups with psychiatry staff; see Additional file 3 for quality criteria assessment; and see Additional file 4 for the Consolidated Criteria for Reporting Qualitative Research (CoreQ) checklist.

### Participants

The interview participants came from a group of respondents to a national survey [24] where. In the individual interviews, participants consisted of 4 psychologists, 5 nurses, and one mental health worker. In the focus group interviews, 2 were psychology students, 6 psychologists, 1 chief psychologist, 2 physicians, 1 assistance physician, 2 mental health workers, 2 nurses with specialist training in psychiatry, 7 nurses, 2 social workers, 1 psychotherapist and due to technical problems, there is no job description for one of the participants in one of the focus groups. No questions were asked in the interviews about the number of years of experience in psychiatry, but information from the national survey may give an approximation of the range of experience; over 60% had more than 10 years of experience working in psychiatry, over 15% had between 6 and 10 years’ experience and over 20% had less than 5 years’ experience [24]. Among participants in the individual interviews, 70% were women, and so were the majority of focus group participants, where 77% were women. A total of 10 individual interviews were conducted, two of which were pilot interviews, where one interview was with a clinical staff member and the other was with a clinic manager. The focus groups included between 5 to 7 participants with a total of 17 participants.

### Data analysis

#### Overview

The interviews were digitally recorded and transcribed verbatim by a professional transcription service. The data were then analyzed according to a phenomenological hermeneutic process, whereby analysis was initiated with a naive reading. Each interview was listened to several times to achieve an understanding of what was said, in parallel with perusal of the transcription. When all interviews had been carefully perused, a *naive understanding* was recorded in writing. The analysis continued with a *structural analysis* of the interviews, a method that can be seen as an oscillation between the text as a whole and the parts of the text. The themes and sub-themes that emerged during the structural analysis were then reflected on in relation to the naïve understanding, as a way of validating the results; the results of this reflection are reported in the discussion section, as the *comprehensive understanding*.

#### Specific procedure for the structural analysis

Meaningful units were picked out which were subsequently condensed into shorter sentences, prior to being further shorted into abstractions. EP wrote the naïve understanding and AT and AHB contributed by listening and reflecting. When all meaningful units had been selected and the condensations formulated, they were printed out on paper. Units and condensations were then cut out and placed on a large table where authors EP and AT identified which condensations resembled each other, after which they were condensations merged into themes and subthemes. This procedure was repeated on several occasions until final themes and subthemes could be determined. These were then compared with the naive understanding [25].

## Results

The results present an incorporated interpretation of the results from the analyses of the individual interviews as well as the focus group interviews.

### Naïve understanding

Caring for patients in psychiatry with SUD was perceived by participants as difficult. They felt that their lack of resources and knowledge was an obstacle in the work. Providing patients with the care they needed was difficult when care for patients' conditions was divided between psychiatry and addiction. It was not uncommon that this meant difficulties in cooperating, even though there was a positive approach to collaboration meetings and, in several cases, structured procedures were available to guide such collaboration. However, it was understood that collaboration evolved with time and could improve. Being able to offer patients more help for substance use issues than what is available today was perceived as a good thing.

### Structural analysis

Findings from the structural analysis are presented within three themes which illuminate the health professionals’ experience of encountering patients in psychiatry with SUD. The themes, each with sub-themes that emerged from the structural analysis, are presented in Table 1. Table 2 shows the detailed flow of the structural analysis from Meaning Units to Themes.

**Table 1:** Overview of themes and sub-themes

Theme	Sub-theme 1	Sub-theme 2
Bridging the organizational gap	Having an established collaboration	Facing difficulties in the collaboration
Having beliefs about the patients you encounter	Working with patients who are exposed to prejudicial thoughts	Expressing prejudicial thoughts towards the patient
Striving to achieve a therapeutic alliance	Having a feeling of developing together	Supporting the patient towards recovery

**Table 2:** Example of the structural analysis from meaning units to themes

Meaning unit	Condensation	Abstraction	Sub-theme	Theme
“And what we also tried to have as a routine is to always have contact with addiction care”	We have tried to have as a routine always to have contact with addiction care	Tried to have a routine always to have contact with addiction care	Having an established collaboration	Bridging the organizational gap
“The process of getting people to collaborate and find collaborative alliances and forms, it is a huge process, it takes almost ten years, before things come into place and then it is time to demolish something”	The process of getting people to collaborate and find collaborative alliances and forms, it's a huge process, takes almost ten years, before things get in place	Huge process of getting people to collaborate, finding collaborative alliances and forms	Facing difficulties in the collaboration	Bridging the organizational gap
“I assume that people tell the truth until the opposite is proven and really it is not, it is a waste of time if the patient is not honest, but sometimes people struggle with these great difficulties with feelings of shame and guilt and then you have to work with it”	It is a waste of time if the patient is not honest, but sometimes people struggle with these great difficulties with feelings of guilt and shame and then you have to work with it	Waste of time in case of dishonesty – encountering difficulties with guilt and shame	Working with patients who are exposed to prejudicial thoughts	Having beliefs about the patients you encounter
“How do you know what the truth is, it is the patient who is the one who responds?”	How do you know what is true, it is the patient who responds?	How do you know that the patient responds truthfully?	Expressing prejudicial thoughts towards the patient	Having beliefs about the patients you encounter
“I think it is probably very individual how to handle it, partly I think that it may also differ between us therapists, how we look at it and also how the doctors look at it, but I can only speak for myself that when I discover that you do not get anywhere in the treatment and you understand that there is more alcohol here than you have been able to map from the beginning”	Very individual how to handle it, it can differ between therapists and also doctors. Can only speak for me, that when I discover that you do not get anywhere in the treatment and you understand that here is more alcohol than you have been able to map from the beginning	Very individual how to handle it, can differ how between therapists. More alcohol than you have been able to map from the beginning when you do not get anywhere in the treatment	Having a feeling of developing together	Striving to achieve a therapeutic alliance
“I’m thinking about those that I have contacted, as it’s been so mostly that I offer to accompany them there, for example to the first physician appointment there, or to a nurse [appointment]…..One begins to build up something so that the patient knows where it is, what the building looks like and so forth, for there is a lot of fear in this too, and resistance.”	I offer to come along for the first conversation with the doctor and nurse, you start to build something so that the patient knows where it is, how the premises look, because there is a lot of fear in this also, and resistance	Offers to accompany to the first visit, so the patient knows where it is, how the premises look, there is a lot of fear in this and resistance	Supporting the patient towards recovery	Striving to achieve a therapeutic alliance

#### Theme: Bridging the organizational gap

The experience of encountering patients with SUD meant bridging the organizational gap and was illuminated by the following two sub-themes: (1) *Having an established collaboration* (2) *Facing difficulties in the collaboration.*

*Having an established collaboration* meant working in an integrated manner, for example through staff from different clinics working together, in the same direction. In the informants’ efforts to provide the patients with healthcare according to their needs, working in an integrated manner was experienced as very important.

”Treating addiction problems [requires] very good collaboration with addiction specialty care, since that is the organization we have in the county.” Manager 7:1b

Having an established collaboration also meant being able to see opportunities and being able to find solutions. This was shown when the staff adapted to the reality they were in, where different clinics had different resources to care for the patients. They could then find a way to overcome the barriers that arose. A prerequisite for being able to provide good care was, however, that collaboration between the units worked well and that there were flexible staff members who were willing to collaborate.

”And the routine we have always tried to have is to have contact with addiction specialty care. So as soon as addiction problems are identified…we contact addiction specialty care. This is a routine we have had to try to collaborate with them and this is also something that has not worked very well, as we experience it. Sometimes they give our patients appointments, after we have referred them, but there is not much collaboration and if the patient doesn’t show up, what they often do is set in different control measures like going and testing oneself, and if the [patient] doesn’t do that, doesn’t show up, it can take time before we get to know about it and sometimes, yeah.” Manager 2:18.

Having an established collaboration meant having the patients’ perspective. This emerged when the informants expressed a wish to collaborate more and work more closely together, by means of having joint meetings where the caregivers together with the patient planned the patient's care in order to reach the patient's goals. The purpose here was that the patient would benefit from better collaboration between psychiatry and addiction care.

”And this has been…very good, when one can sit down together and plan collaboratively, when the patient provides their consent; this is often very effective.” Focus group 13:26

Bridging the organizational gap meant *facing difficulties in the collaboration*. Facing difficulties in the collaboration meant that responsibility was divided rather than shared. This was illuminated by a complexity in the collaboration. Not knowing what the other unit would offer the patient made the contact with the patients harder. It was important to know exactly which task each unit had in order to facilitate collaboration. Not being aware of the task resulted in speculations about the partners in collaboration instead of knowing that collaboration was part of the mandate.

”So a concern I think we have here is that addiction specialty care is so terribly unpredictable. We don’t have the faintest idea what we will get.” Manager 3:29

Facing difficulties in the collaboration also meant being unsure of one´s own task. Feeling unsure of what was included in one’s professional tasks was perceived as difficult. This was shown in the analysis when the patients who were perceived as difficult were described as being referred to other clinics.

”I think that if one has an [ongoing] treatment [for]…CBT, social phobia or something else, and [addiction problems] suddenly turn up and one doesn’t know, how should one address this? And then it’s easiest to just send a referral to addiction specialty care so one gets rid of the patient and the problem, but actually one hasn’t helped anyone.” Manager 3:62

Facing difficulties in the collaboration also meant struggling to make it work even though it sometimes felt hopeless. Seeing the possibilities without being able to directly influence the actual care given the patient could trigger a feeling in informants of being stuck in a treadmill. This was illuminated by a desire in the respondents to make a change and to find a way to improve their daily work.

“There’s something wrong with the way it is split up, that…we don’t have the training, we don’t generally have the knowledge….and it’s another unit [that is supposed to give that care]. Clearly the collaboration can be improved, but it still ends up being a catch-22 for the patients…. Do you understand what I mean? Referral here, referral there, it’s ridiculous, really.” Manager 1:21:10

#### *Theme:* Having beliefs about the patients you encounter

The experience of encountering patients with SUD meant having beliefs about the patients one encounters, illustrated by the sub- themes: (1) *Working with patients who are exposed to prejudicial thoughts* (2) *Expressing prejudicial thoughts towards the patient.*

*Working with patients who are exposed to prejudicial thoughts* meant having confidence in them. This was expressed by staff who listened to the patients and tried to help them to be able to tell their life stories without feelings of guilt or shame.

“My starting point is that people are speaking the truth until the opposite is shown, and really it’s not like that; yeah, it’s a “waste of time” [sic] one could say, if the patient is not honest, but sometimes people are struggling with these huge difficulties, with feelings of shame and guilt, and then one has to work with that.” Manager 6:64

Working with patients who are exposed to prejudicial thoughts meant facing the patients’ fears. This was illuminated by the patients not wanting to admit that their addiction problem because it felt easier to attend psychiatric care than addiction care. It was perceived to be less shameful to have an ongoing contact with psychiatric care.

“I think there is stigmatization also because one doesn’t want to identify with very run-down, social outcast alcoholics, it can also be that.” Manager 10:18

Working with patients who are exposed to prejudicial thoughts meant wanting to free the patient from feelings of guilt and shame. This was expressed by staff wanting to find a new solution for patients to get the help they need, in a way that reduces feelings of guilt and shame. By offering digital interventions they saw a possible way of getting past the stigmatization that the patients experience, making talking to patients about their substance use into a less delicate subject with a digital tool as a help.

“And [digital interventions are] more about being engaged oneself, in a way that is also free of guilt and shame…because they are not alike, it’s not the same thing to be exposed to others’ values or judgements in the same way [as in face-to-face meetings]….” Manager 10:55

*Expressing prejudicial thoughts towards the patient* meant feeling clueless about the patient´s level of actual substance use. This was illuminated by the informants who felt that patients seldom reported their substance use at all, or under-reported it. The informants felt that they needed to detect the use themselves, which was difficult since the patients often were very inventive when they were actively using alcohol or drugs and that although staff were experienced, they still could miss that the patient had an ongoing use.

”In my experience [patients] are not so [forthcoming], so it’s more of an understatement when one asks about drugs, or alcohol and drug use…[The patients] are very different but there are many who minimize [their use] or say [that it’s] less than what it actually is.” Manager 7:5

Expressing prejudicial thoughts towards the patient meant working erratically and was experienced as not being able to provide equal care to the patients. This was expressed in relation to clear guidelines regarding basic assessment at intake in psychiatry, that applied only to newly admitted patients. For patients who were already admitted to the unit, there were no guidelines regarding the basic assessment. Staff assumptions regarding SUD were based on the symptoms or difficulties that the patients presented when discussion between the professional and the patient took place regarding further action on possible treatment of the substance use. For example, not all patients were asked about their substance use. The decision whether or not to obtain more information about substance use depended on how much information the staff already had.

”Yes, we administer the AUDIT to all, I would say that, but the DUDIT I don’t think we administer to all [patients] – [we do that] only if one suspects that there is some [drug use].” Manager 6:14

Expressing prejudicial thoughts towards the patient meant working with preconceived perceptions of gender. This was expressed by informants who saw a difference between women and men in their substance use. The difference concerned both how quickly the substance use became problematic for the individual but also how it affected the family.

“And women become alcoholized much more quickly than men, they become impulsive in a way that it not good and which they themselves also are very affected by, for example getting far to angry at their kids or whatever. And behind this there is an alcohol consumption that is too high. Too much wine, quite simply, is what I think I often see.” Focus group 13:39

#### Theme: Striving to achieve a therapeutic alliance

The experience of encountering patients with SUD meant striving to achieve a therapeutic alliance and was illustrated by the sub- themes: (1) *Having a feeling of developing together* (2) *Supporting the patient towards recovery.*

*Having a feeling of developing together* meant being on a journey with the patients towards their recovery. This was illuminated by the finding that the informants and the patients are successively getting to know each other during the ongoing contact. This also meant that the informants’ knowledge of the patient gradually increased when the staff gained a deeper understanding of the patient.

“I think like this, that even if it’s not obvious from the beginning when one encounters the patient that they have a drug problem, it usually emerges over time.” Manager 6:1b

Having a feeling of developing together also meant trying to convey the seriousness of the situation. This was expressed by the informants describing it as difficult to tell patients that they should not drink alcohol, and the patients thinking they had to abandon their social life on the weekends completely when the informants encouraged patients to be completely sober.

“So I think many don’t really take this in, or those that work and accompany their chums somewhere, ‘we’ll have a drink’…and that’s where they start, and they can’t really see the consequences.” Focus Group 12:28

Having a feeling of developing together also meant being tentative. This was expressed by informants taking it step by step and working with the patient’s motivation. The informants took into consideration that the contact with them was voluntary for patients and that it was meaningful for the patients to maintain the contact.

”So part of the model is that one gets a little motivation for treatment, and then one works with trying to increase [that motivation].” Manager 3:3

Striving to achieve a therapeutic alliance meant *supporting the patient towards recovery*. It meant being available for the patient. This was illuminated by the informants during a certain period relieving the patient from taking their own responsibility and instead carefully guiding the patient to be able to get more of the care offered. This was for example expressed when the informants accompanied patients to external clinics to support them, when the patients found it difficult to make the first contact with the new clinic.

“I’m thinking about those that I have contacted, as it’s been so mostly that I offer to accompany them there, for example to the first physician appointment there, or to a nurse [appointment]…..One begins to build up something so that the patient knows where it is, what the building looks like and so forth, for there is a lot of fear in this too, and resistance. It’s about change and…one doesn’t know how long it will last, but it has lasted a bit longer if one has a contact [with the external clinic] and I think that one would need more, really, collaboration, and meeting together with them than what we already do perhaps.” Focus group 12:53

## Discussion

This study contributed findings based on content and phenomenological-hermeneutic analysis of interviews with unit managers for psychiatric outpatient clinics as well as focus group interviews with staff working in psychiatric outpatient clinics. The study provides insight into how staff are struggling to help patients progress in their treatment and reach their treatment goals.

The comprehensive understanding of the results, presented here, is the interpreted whole of the present study. Staff experiences of encountering patients with SUD were illuminated based on three themes: bridging the organizational gap, having beliefs about the patients you encounter and striving to achieve a therapeutic alliance. Taken together the results of the analysis could be interpreted as that the staff are alternating between extremes and struggling to provide the care that the patients need. This was shown when the staff described collaboration as a state of “either/or”: things are good when collaboration exists, but when it does not, there is a deficit and this makes the work difficult. The findings also imply that on one hand the staff are striving to achieve a therapeutic alliance, and on the other hand having prejudicial beliefs about the patients they encounter.

Structural barriers, such as that psychiatry and social services can both have joint responsibility for patients with comorbidity, led to difficulties in collaboration with addiction care. Other structural barriers could occur when there was uncertainty about one's own or others' tasks, together with a lack of clarity and unpredictability between organizations, reflected in the sub-theme difficulties in collaboration. On the other hand, we found that when the staff had overcome the structural barriers, the collaboration worked much better. This can also be interpreted as important for the patient's well-being, as shown in a study where the patient's feeling of security was affected by how staff communication with another care provider was experienced; the patients’ stress decreased when they did not have to repeat themselves at the new care center [26]. Other research has found that collaboration based on mutual partner trust is required, rather than the expectation of receiving something in return from the collaboration [27].

Having SUD and seeking help for it can often be experienced as difficult for the patient [28, 29]. Previous research has shown that for men with substance dependence, the experience of being validated, in that someone saw their suffering, made them feel relaxed [30]. Our results suggest that the experience of suffering from prejudicial thoughts and dealing with feelings of guilt and shame could lead to obstacles for patients who dared to tell staff about their situation. Previous research has found that when patients experienced prejudicial thoughts or stigmatization from healthcare staff, both directly and indirectly, they felt that they were put into stereotypical categories and that that the staff behaved and spoke to them in a degrading and patronizing manner [31]. Also, ignorance of how to treat of this patient group leads to prejudice and stigmatization [28]. Our results suggest that stigma and feelings of guilt or shame seem to be an obstacle for patients in telling their healthcare providers about their situation. This concords with an earlier study showing that the feeling of being someone who creates problems for other persons in their surroundings also affects the motivation to seek help [32]. Such barriers to seeking and accessing SUD treatment could be overcome by offering remote or digital addiction treatment. One recent systematic review on computer-based interventions for patients with symptoms of SUD and mental health issues show an improvement in mental well-being and substance use compared with a group that were allocated to a waiting list and received psychoeducation. The review also showed that computer-based interventions offered with therapist support (guided intervention) were even more effective than unguided computer-based intervention [33]. With complementary digital interventions, patients could maintain their contact with their outpatient psychiatric clinic and at the same time receive treatment for SUD via the internet. This could contribute to overcoming the barriers where there are difficulties in having parallel contacts with different healthcare services. Implementing such a structure could contribute clarity regarding which clinic does what and what the division of responsibilities is between the clinics, potentially benefiting collaboration between psychiatry and addiction services.

Making sure that the patient participated, when good collaboration between psychiatry and addiction clinics was present, was seen as a prerequisite for providing good healthcare; this emerged in the sub-theme of having an established collaboration. In the present study, the patient’s motivation to deal with their SUD was sometimes perceived as lacking or limited or, sometimes, as high. Earlier research has found that it is very important to be able to motivate patients through conversations and stimulate initiation of a change process [34]. Participation in healthcare also contributes to patients feeling more satisfied and secure in their treatment [35, 36].

On the one hand, encountering patients with SUD meant working with patients who are struggling not to be labeled as addicts. This was shown in the sub-theme for working with patients who are exposed to prejudicial thoughts. On the other hand, the sub-theme of expressing prejudicial thoughts towards patients showed that staff were sometimes contributors to discriminating against patients by showing distrust and having preconceived notions. A previous study has shown that there are staff in the healthcare system who think that addiction is a self-inflicted disease and that patients with substance dependence are not really ill. This, in turn, increases the stigma of an already vulnerable patient group [28]. While the present study shows contradictions in the staff’s perceptions of patients, it also shows that they are trying to establish an alliance with the patient in an attempt to achieve a therapeutic result. They work with a group of patients who are often stigmatized at the same time as they can contribute to stigmatization, often unconsciously. Patients who express a higher degree of self-stigma have been found to often experience reduced satisfaction and trust for healthcare services [37]. Therefore, it is important that staff work with their own perception of patients' perceived willingness to undergo treatment for alcohol dependence [38], through training and supervision [20].

The road to recovery from SUD was interpreted as a journey with the patients, where the staff learned more about the patient's individual needs during the journey, and it was important to be available and sensitive to the patient's needs. Staff may experience a lack of knowledge about caring for patients with SUD [39], and it is also important for staff to be aware that patients' lives can change rapidly and lead to alienation and self-destruction [40]. Lack of explicit awareness can contribute to our finding that staff in the present study struggled with the experience that in certain situations they were deceived by the patients and felt they had to search for the truth and try to help the patient realize the seriousness of their SUD. This can be interpreted from a life world perspective, where the world can be interpreted as mutual for all people, but it does not mean that all people have the same view or have the same relationship to the world [41]. In relation to the results of the present study, where the path to recovery was seen as a journey together with the patient, and the alliance between the patient and the care staff developed over time, the relationship between staff and patients was affected by different views of the world. Patients experience a world of life that is affected by wanting to continue their substance use or, for those who want to change their behavior, the existence of structural barriers regarding their care.

This study had both strengths and limitations. Its major strength was illumination of the struggles staff encounter when working with patients with substance use within the outpatient psychiatric context, an area much discussed among clinicians in addiction settings, but seldom penetrated in depth. The phenomenological method also contributed to a flavorful depiction of nature of staff’s day-to-day struggles. Also, the purpose of the study was to further explore challenging issues when treating patients in psychiatry, a topic previously explored in our quantitative survey [24]. The study’s primary limitation was possible bias, in that participants who were approached for an interview or focus group had already indicated their interest in working with digital interventions within psychiatry. Nonetheless, we perceive that the results may be generalizable to contexts where interest in digital interventions is not necessarily high. The results could also have been strengthened by triangulation, where observations from the interviews would have been noted and analyzed in addition to the individual interviews and focus group interviews.

## Conclusion

The study shows that collaboration between different clinics is sometimes perceived as a barrier for patients to seek care. We conclude that an opportunity for patients to receive digital treatment for SUD while remaining in their regular psychiatric clinic could build a bridge between psychiatry and addiction care and in this way, might to some extent reduce the gap between the psychiatry and addiction care. Patients who have not wanted to be referred to addiction care could log in to the digital treatment from home and in this way, we could increase the proportion of patients who seek help and receive treatment. However, establishing such an infrastructure should be seen as a complement to regular addiction care as not all patients would want treatment through digital interventions.

## Supplementary Information

**Additional file 1.** Interview guide: individual interviews with clinic managers in psychiatry.

**Additional file 2.** Interview guide: psychiatry focus group.

**Additional file 3.** Quality criteria.

**Additional file 4.** COREQ checklist.

## Data Availability

The datasets used and/or analyzed during the current study are available from the corresponding author on reasonable request.
