# Supplementary material for: Staff experiences of encountering and treating outpatients with substance use disorder in the psychiatric context: a qualitative study
Source: Addict Sci Clin Pract. 2021 May 10;16:29. doi: 10.1186/s13722-021-00235-9 (PMC8112046; doi:10.1186/s13722-021-00235-9)
Supplement: Supplementary file 1 — Additional file 1. Interview guide: individual interviews with clinic managers in psychiatry. [file 13722_2021_235_MOESM1_ESM.docx]

Interview guide for the individual interviews with psychiatry clinic managers

[ The interview was carried out in preparation for a possible coming collaboration on a pilot study.]

1. Please tell us your name, and your profession, where you work and what tasks you have, both at your clinic and any other relevant workplaces / clinics.

2. Do you experience that patients’ alcohol and drug habits affect their treatment at your clinic? If so, how?

3. Screening: Refer to the survey results (1)[about guidelines/preliminary results from the survey] and ask: How is screening carried out at your clinic? How (well) does it work?

4. Brief interventions (BI). In the survey, we asked about the guidelines for BI (reproduce preliminary results from the questionnaire) ^[[1]](#footnote-1)^. How is BI carried out at your clinic? How (well) does it work?

5. If you collaborate with the addiction care, how does it work?

Does you / your clinic need to improve / change methods for screening and short interventions for patients' alcohol and drug habits? If so - what is missing now and what is needed in the future?

**Give the information about the digital interventions that could be offered in a stepped care model.** Inform the participants that digital interventions with stepped care are a concept that aims to offer a flexible treatment instrument: a framework for different types of alternatives, which makes it possible for both treatment provider and patient to choose the option or alternatives that work best. Anne's research group [senior author] is working to develop the digital intervention concept so that it can be used in psychiatry.

**Show and tell the participants about the stepped care pictures - give them a copy**

7. What do you think about the concept of digital stepped care, now that I've told you about it? What do you think / feel about the different interventions? Which ones would you benefit from and why?

Based on the stepped care images: Reflect freely on components, order and context.

8. Do you see any benefits in using such a concept? If so, please describe.

9. Do you see any disadvantages in using such a concept? If so, please describe.

10. What do you think could facilitate implementation in your clinic? What obstacles do you see in implementing the concept in your clinic? How can these obstacles be addressed?

11. Do you think such a digital stepped care concept can be effective? How? Why not?

- For example: influence the patient's alcohol and drug habits, mental health, physical health, treatment and / or therapists, the treatments (eg streamlining treatments, shorter treatment time etc / time + resources = financially streamlining)

12. To plan a pilot study in the autumn of 2015 on the digital stepped care concept, we would like to carry out focus groups with care staff in the autumn of 2014. Could we carry out a focus group with 5 - 7 of the treatment staff at this clinic this autumn?

13. Does your clinic have the opportunity and interest to participate in the upcoming pilot study in the autumn of 2015?

14. Do you have any questions?

References

1. Sundström C, Petersén E, Sinadinovic K, Gustafsson P, Berman AH. Identification and management of alcohol use and illicit substance use in outpatient psychiatric clinics in Sweden: a national survey of clinic directors and staff. Addiction science & clinical practice. 2019;14(1):10.

1. BI is a brief intervention for problematic alcohol- or drug use, with the purpose of motivating the patient to take the first step towards change. [↑](#footnote-ref-1)
