# Supplementary material for: Staff experiences of encountering and treating outpatients with substance use disorder in the psychiatric context: a qualitative study
Source: Addict Sci Clin Pract. 2021 May 10;16:29. doi: 10.1186/s13722-021-00235-9 (PMC8112046; doi:10.1186/s13722-021-00235-9)
Supplement: Supplementary file 2 — Additional file 2. Interview guide: psychiatry focus group. [file 13722_2021_235_MOESM2_ESM.docx]

Interview guide for focus groups with psychiatry staff

1. Presentation round. Please, tell us your name and what working tasks you have at the clinic.
   1. Presentation of digital Interventions: What are digital interventions? Present the purpose with the interview.
2. Do you feel that patients' alcohol and drug habits affect your treatment? If so, how?
3. Do you carry out alcohol and drug screening? How does it work?
4. What do you do when patients have alcohol and / or drug problems? (do you refer them to an addiction clinic, or do you offer and carry out BI at your clinic)
5. How does the collaboration with addiction care work? (psychiatry – addiction care – patient)
6. Do you think that your clinic would need to develop / change your methods for screening and treating patients' alcohol and drug habits? Why / why not?

**Provide information on the digital interventions that could be offered in stepped care.** Digital interventions with stepped care are a concept that aims to offer a flexible treatment instrument. The patient is referred to evidence-based interventions that are offered on secure sites where the patient can screen / test his or her alcohol and drug habits, get advice and also receive treatment. This is a framework for different types of options, it enables the therapist and patient to choose the option(s) that work best. Anne's research group is working to develop the digital intervention concept so that it can be used in the psychiatric care. **Show and tell them about the stepped care pictures - give the participants a copy**

1. What do you think about the concept of digital stepped care, now that I have told you about it? What do you think about the different interventions? Which one would you benefit from and why?
2. Do you see any benefits in using such a concept? Which benefits?
3. Do you see any disadvantages in using such a concept? Which disadvantages do you see? (It is important for us to know if there is something that can be negative, so that we can take it into account when developing an implementation model.)
4. How do you feel / think about referring the patient to a digital service for screening? Feel free to imagine that you have a patient in front of you who may have alcohol / drug problems. How could you use a digital service? Please describe in detail how you would proceed in the meeting with the patient.
5. How do you feel / think about referring the patient to a digital service for short intervention? How would you like to follow up on how the patient is doing?
   1. If the digital service could be guided: What are your thoughts about serving as a guide to the patient in the treatment via the internet^[[1]](#footnote-1)^ (after a short training)?
   2. Imagine that you have a patient in front of you who has alcohol / drug problems. How would you use a digital service to help the patient? Please describe in detail how you would proceed in the meeting with the patient.
6. How would you like to follow up on how the patient is doing with the digital service?
7. What do you think could facilitate the implementation in your clinic? What obstacles do you see in implementing the concept in your clinic? How can these obstacles be addressed?
8. If you had the opportunity to refer the patient to such an internet-based service, how do you think it would affect your work / treatment activities?

1. In ”blended” format or via secure messages, chat, telephone etc. [↑](#footnote-ref-1)
