# Supplementary material for: Staff experiences of encountering and treating outpatients with substance use disorder in the psychiatric context: a qualitative study
Source: Addict Sci Clin Pract. 2021 May 10;16:29. doi: 10.1186/s13722-021-00235-9 (PMC8112046; doi:10.1186/s13722-021-00235-9)
Supplement: Supplementary file 3 — Additional file 3. Quality criteria. [file 13722_2021_235_MOESM3_ESM.docx]

Quality criteria assessment

This study was based on individual interviews as well as focus group interviews with both managers and staff in the psychiatric outpatient context. To maintain reliability and validity, we choose to follow quality criteria (credibility, transferability/generalizability and dependability) for qualitative studies (1). Credibility was achieved through a varied selection of informants in the study, both managers in different professions and through focus group interviews with staff from three different units. The interviewers and the authors who analyzed the material all have a clinical background, which strengthens the researchers' credibility. To validate transferability/generalizability, the results of the analysis were presented continuously in the research group during the analysis process. Dependability can be described as data stability over time. After the initial interviews with a manager and a staff member, conducted by author AHB (associate professor, licensed clinical psychologist, PhD), all interviews with managers were conducted by Ann-Sofie Bakshi, PhD. Focus group interviews were conducted by Kristina Sinadinovic, PhD, Christopher Sundström (psychologist, PhD-student) and AHB. All interviews were recorded and transcribed verbatim. During the course of the analysis, the material was analyzed by author EP (reg. nurse, PhD-Student), who then presented the ongoing analysis material for Anna Thurang, PhD (registered nurse) and AHB. The findings were discussed on several occasions from several different perspectives. The findings of the study may be seen as an interpretation of what emerged in all the interviews. The interpretations have not been directly fed back to the informants. None of the interviewers had any prior relationship to the participants. All interviews started with a presentation of the interviewers. To strengthen the reliability of the study, the researchers have been inspired by COREQ and by following this 32-item checklist, replicability increases (2).

References

1. Polit DF, Beck CT. Nursing research: Generating and assessing evidence for nursing practice: Lippincott Williams & Wilkins; 2008.

2. Tong A, Sainsbury P, Craig J. Consolidated criteria for reporting qualitative research (COREQ): a 32-item checklist for interviews and focus groups. International journal for quality in health care. 2007;19(6):349-57.
